# Integrin signaling downregulates filopodia during muscle–tendon attachment

**DOI:** 10.1242/jcs.217133

**Published:** 2018-08-16

**Authors:** Benjamin Richier, Yoshiko Inoue, Ulrich Dobramysl, Jonathan Friedlander, Nicholas H. Brown, Jennifer L. Gallop

**Affiliations:** 1 The Gurdon Institute, Tennis Court Rd, Cambridge CB2 1QN, UK; 2 Dept. of Biochemistry, University of Cambridge, Cambridge CB2 1GA, UK; 3 Dept. of Physiology, Development and Neuroscience, University of Cambridge, Cambridge CB2 3DY, UK

**Keywords:** Filopodia, Integrin signaling, Actin dynamics, Muscle, *Drosophila*

## Abstract

Cells need to sense their environment to ensure accurate targeting to specific destinations. This occurs in developing muscles, which need to attach to tendon cells before muscle contractions can begin. Elongating myotube tips form filopodia, which are presumed to have sensory roles, and are later suppressed upon building the attachment site. Here, we use live imaging and quantitative image analysis of lateral transverse (LT) myotubes in *Drosophila* to show that filopodia suppression occurs as a result of integrin signaling. Loss of the integrin subunits αPS2 and βPS (also known as If and Mys, respectively, in flies) increased filopodia number and length at stages when they are normally suppressed. Conversely, inducing integrin signaling, achieved by the expression of constitutively dimerised βPS cytoplasmic domain (diβ), prematurely suppressed filopodia. We discovered that the integrin signal is transmitted through the protein G protein-coupled receptor kinase interacting ArfGAP (Git) and its downstream kinase p21-activated kinase (Pak). Absence of these proteins causes profuse filopodia and prevents the filopodial inhibition mediated by diβ. Thus, integrin signaling terminates the exploratory behavior of myotubes seeking tendons, enabling the actin machinery to focus on forming a strong attachment and assembling the contractile apparatus.

## Abstract (precis)

**Summary:** Integrins signal through Git and Pak to downregulate filopodia when muscles reach their target attachment site in *Drosophila*.

## INTRODUCTION

**Fig. 1.**
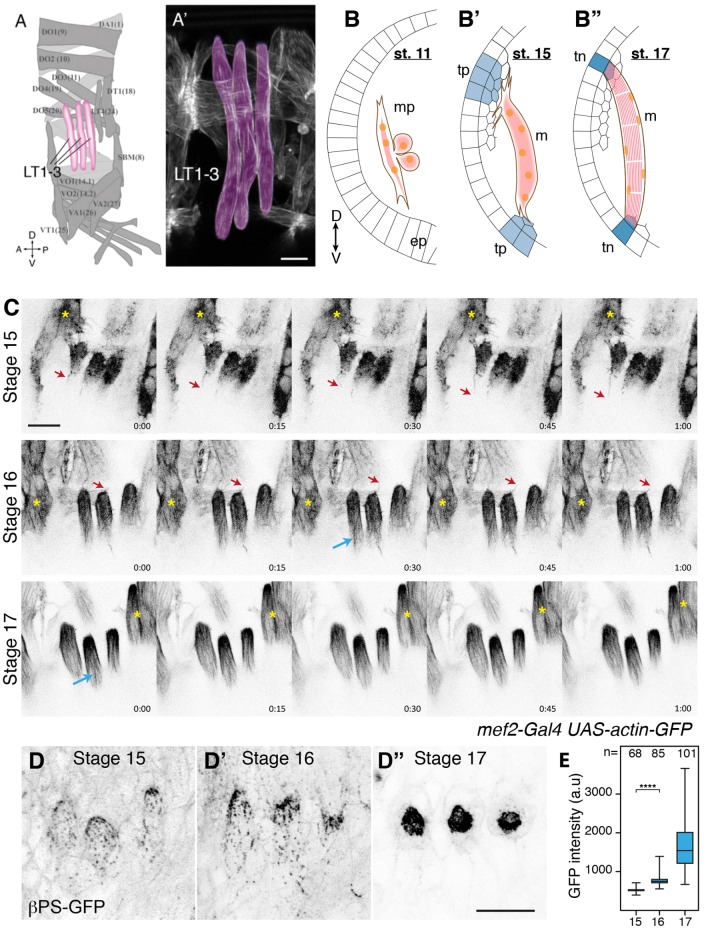
**Filopodia dynamics in embryonic LT muscles.** (A,A′) Schematic of the stereotypical muscle pattern in segments A2 to A7 (Ruiz-Gómez et al., 1997). Magenta, lateral transverse muscles (LT1–3); light gray, interior muscles; gray, other muscles. (A′) Mature LT muscles (magenta) in a stage 17 embryo visualized by *mef2-Gal4 UAS-GFP-actin*. (B–B″) Schematic of developmental steps of *Drosophila* myogenesis (embryo cross-section). (B) Stage 11: fusion-competent myoblasts fuse to one founder cell to form a multinucleate myotube progenitor (mp). (B′) Stage 15: the leading edge of extending myotubes form filopodia, searching for their targets, the tendon precursors (tp) located in the epithelial layer (ep). (B″) Stage 17: filopodia formation ceases, and both ends of the myotube attach to terminally differentiated tendon cells (tn). (C) Time-lapse images of LT myotube tips at stages 15, 16 and 17. Filopodia form during stage 15 and 16 but appear smaller at later stages (red arrows). Within the myotube, actomyosin fibers develop during stage 16 (blue arrows). Yellow asterisks, adjacent myotubes. Time is given in min:s. (D–D″) βPS–GFP expressed at endogenous levels, revealing integrin accumulation at the muscle attachment site at stages 15, 16 and 17. (E) Quantification of the βPS–GFP intensity at myotube tips (8–10 embryos). The box represents the 25–75th percentiles, and the median is indicated. The whiskers show the range. ****P=2.2×10^−16^ (Mann–Whitney test). Orientation is as indicated in A and B [dorsal (D), ventral (V), anterior (A) and posterior (P)]. Scale bars: 10 μm.

Muscle development involves similar phases of differentiation and movement in *Drosophila* and vertebrate embryos (Schweitzer et al., 2010). *Drosophila* somatic muscles display a stereotyped pattern of 30 muscles per abdominal hemisegment (Fig. 1A,A′). The lateral transverse (LT) muscles migrate and elongate towards the epidermis, and their attachment sites, near the surface, are particularly accessible for live imaging (Fig. 1A,A′). Each LT muscle is a single syncytial cell, the myotube, which is formed by fusion of fusion-competent myoblasts to a founder cell (Rochlin et al., 2010). Each founder cell contains the transcriptional instructions for the development of that particular muscle. This includes the number of fusion events and the sites of attachment to the epidermis (Fig. 1B–B″) (de Joussineau et al., 2012). While some of the guidance cues known to target myotubes to their tendon cells overlap with those guiding neuronal cell migration (Slit, Robo and Derailed), some cues are unique to specific myotubes, such as the transmembrane protein Kon-tiki in ventral-longitudinal muscles (Callahan et al., 1996; Estrada et al., 2007; Kramer et al., 2001; Schnorrer et al., 2007). Myotube migration and tendon cell specification proceed in concert to organize muscle architecture (Schweitzer et al., 2010) with, for adult indirect flight muscles, extensive protrusions occurring in both cell types at the time of attachment (Weitkunat et al., 2014).

In the embryo, the myotube ends that move toward the tendon cells produce actin-rich finger-like protrusions known as filopodia (Schnorrer and Dickson, 2004), which have been implicated in the ability of cells to find their correct location (Davenport et al., 1993; Jacquemet et al., 2015). Once at the right locations, the myotubes need to stop migrating, otherwise secure attachments cannot form and myotubes ‘overshoot’ the tendon cells. Such a phenotype has been observed in *Drosophila* deficient in G protein-coupled receptor kinase interacting ArfGAP (Git) (Bahri et al., 2009). At tendon cells, myotubes make a strong integrin-based attachment, protrusions reduce and the myotube tips become rounded. There is concerted assembly of the contractile actin machinery to form the sarcomeres, and tension itself is implicated in this process (Weitkunat et al., 2014). This developmental process illustrates the exquisite intracellular specificity of the actin cytoskeleton, with appropriate actin filament nucleators, elongators and bundlers recruited to different cell areas in a spatially and temporally coordinated manner. Remodeling of the actin cytoskeleton is proposed to occur actively, for example from the activation of Rho-type GTPases or phosphorylation cascades. Within the context of myotube migration, one could also envisage that the building of the extensive contractile machinery of the myotube could lead to a natural reduction in the migratory, protrusive actin activity instead of being actively suppressed at the appropriate time.

In this study, we investigate the role of integrins in the regulation of filopodia during the formation of the muscle–tendon attachment site. We find that, as the muscle reaches the tendon cell, integrins have two important activities: their well characterized role in attaching to the tendon cell via an intervening extracellular matrix, and a new function mediating a signaling pathway that actively suppresses filopodia. We characterize Git and its downstream target p21-activated kinase (Pak) as components of this pathway. The transition from migration to muscle–tendon attachment provides a key example within a whole organism, where the contribution of integrins demonstrably involves signaling in addition to forming adhesions. Thus, integrin signaling helps to provide a migratory stop signal that acts in conjunction with the formation of an integrin adhesion junction at the muscle ends.

## RESULTS AND DISCUSSION

### Filopodia at the myotube leading edges are downregulated when the attachment with the tendon cell is formed

Myotubes migrate towards prospective tendon cells in the epidermis and form stable attachment sites. Contractile actomyosin bundles then generate muscle contraction and embryo movement (Schweitzer et al., 2010) (Fig. 1A–B″). By observing expression of GFP–actin in the dorsal tip of lateral transverse (LT) muscles (using the muscle-specific Gal4 driver mef2-Gal4) in live embryos, we visualized filopodial dynamics (Fig. 1C, red arrows; Movies 1–3). Dorsal LT tips were very close to each other with active filopodia during stage 15 and early stage 16 (∼3 h, Fig. 1C, stage 15). As development progressed, LT tips became more spaced out, and the number and length of filopodia reduced while actin bundles formed instead within the myotube (Fig. 1C, blue arrows), with a rounded end of the myotube making the attachment (Fig. 1C, stage 16). At stage 17, filopodia had become very rare and muscle contractions started (Fig. 1C, stage 17). This apparent reduction of filopodia number at the same time that the attachment forms is consistent with previous observations in embryonic ventral oblique muscles (Bate, 1990; Schnorrer and Dickson, 2004). The integrin expressed by the muscles is the heterodimer αPSβPS, which attaches the muscle to the tendon cell so that the force of muscle contractions is transmitted to the exoskeleton for movement (Maartens and Brown, 2015; Devenport et al., 2007). We observed, similar to what was seen in previous studies in indirect flight muscle development (Weitkunat et al., 2014), that filopodial downregulation and βPS integrin accumulation at the attachment sites occurred simultaneously (Fig. 1D,E).

### Integrin signaling downregulates filopodia

**Fig. 2.**
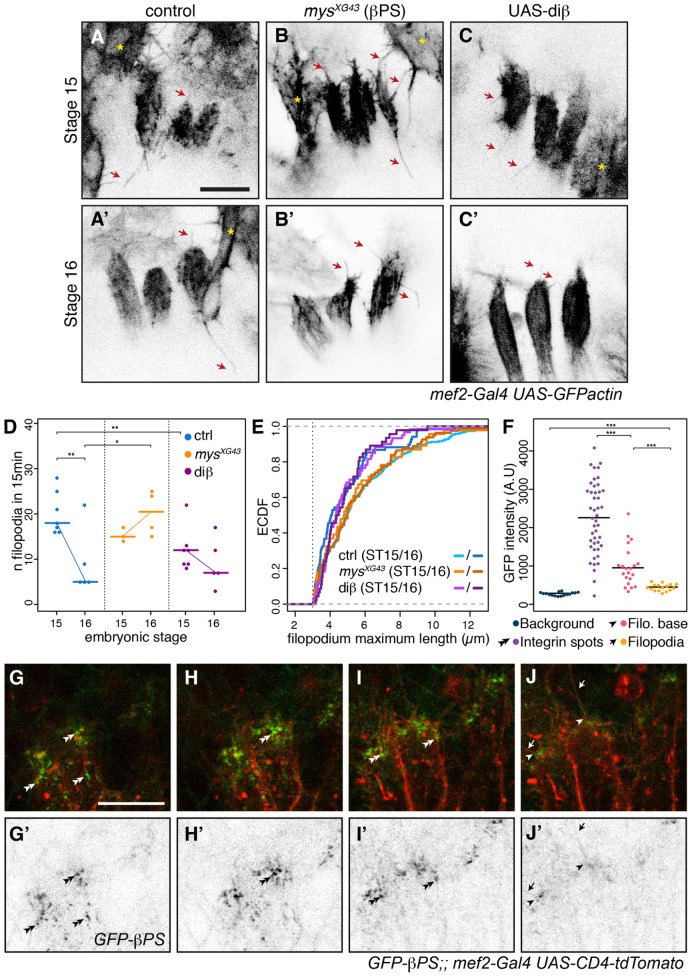
**Integrins regulate filopodia formation at LT muscle tips.** (A–C) Still *z* projections from time-lapse movies showing control (A,A′), zygotic βPS mutant *mys^XG43^* (B,B′) and UAS-diβ (C-C′) LTs at stage 15 and 16. Filopodia are marked with red arrows. Yellow asterisks, adjacent muscles. (D) Number of filopodia at LT tips during initiation and maturation of attachment site in a 15-min time window. Graph showing individual values (dots) and median (horizontal bars). Thin lines between stages illustrate the change in filopodia number. *P<0.025, **P<0.01. (E) We apply a cut-off at 3 µm to discard the shortest structures and show an empirical cumulative distribution function (ECDF) of the maximum length values. Each point on the curve indicates the proportion of filopodia with a given maximum length or less. The curve for ctrl at stage (ST)16 shifts towards the left compared to ctrl at ST15, indicating that the proportion of long filopodia is smaller at stage 16. (F–J′) βPS–GFP in LT tips (stage 15 embryos) in four focal planes (0.5 µm distance) (G–J). βPS–GFP integrin (green) localizes at the membrane (red, *mef-Gal4 UAS-CD4tdTomato*), including within filopodia (arrows), filopodia base (arrowheads) and strongly at focal adhesions (double arrowheads). (F) GFP intensity quantification (*n*=3 embryos). ***P<0.001, ****P<0.0001 (see Table S1). Scale bars: 10 µm.

To test whether integrins are inhibiting filopodia formation, it was necessary to quantify and characterize the filopodial behavior produced by muscles LT1–3 (as shown in Fig. 1A). To do this, we developed an image segmentation and analysis pipeline to reconstruct filopodia in time-lapse movies from confocal microscopy *z*-stacks. The location of LT muscle tips within a complex tissue environment containing other muscles, and the extension of filopodia in many directions, made the analysis too complex for automated quantification software. We therefore employed a semi-automated pipeline consisting of three main steps: (1) automatic segmentation and extraction of filopodia segments, (2) manual editing to remove false detections and merging of segments from the same filopodium, and (3) automated linking of edited filopodia across time points (Fig. S1 shows example fits). The pipeline outputs a list of filopodia and their coordinates for each time point of their existence. The reconstruction of very small filopodia was very noisy; therefore, we excluded them to focus on filopodia that reached a maximum length of at least 3 µm and existed for at least two time frames separated by 15 seconds. We then extracted the maximum length and the average number of filopodia (a *z* projection is shown in Fig. 2A, Movie 1). We quantified the filopodia for the three LTs together, rather than individually, as the three myotube tips are closely apposed and difficult to separate. Because we label all muscles, adjacent muscles (Fig. 2, yellow asterisks) can mask LT muscle tips or their filopodia (Fig. 2, red arrows) in *z* projection images, but the use of original *z*-stacks in the pipeline avoids this issue. Using this method, we validated that a strong reduction in filopodia number occurs from stage 15 to stage 16 in control embryos (Fig. 2D).

With this tool at hand, we visualized GFP–actin in embryos lacking the integrin βPS subunit (*myospheroid^XG43^*, *mys^XG43^*). In *mys^XG43^* mutant myotubes, filopodia were resistant to the downregulation usually observed by stage 16 (Fig. 2A′,B′,D; Movie 4). A similar effect was observed in embryos lacking the integrin αPS2 subunit (*inflated^B4^*, *if^B4^*) although further defects in muscle development prevented accurate reconstruction (Fig. S2; Movie 5). *inflated* is the only αPS subunit expressed in muscles, whereas both αPS1 (encoded by *mew*) and αPS2 are expressed in tendon cells (Devenport et al., 2007). These results suggest that integrins are required within the muscles to regulate filopodia at myotube tips. To distinguish between the signaling and adhesion functions of integrins, we used diβ, a chimeric protein in which the intracellular domain of βPS is fused to the extracellular and transmembrane domains of a constitutively active form of the receptor Torso (Martin-Bermudo and Brown, 1999). This fusion protein constitutively dimerizes the cytoplasmic domain of the βPS subunit, and mimics integrin signaling, even in the absence of integrin-mediated adhesion to the extracellular matrix (Martin-Bermudo and Brown, 1999). The expression of diβ in muscles prematurely reduced filopodia number in stage 15 embryos to a level similar to that seen at stage 16 in control embryos (Fig. 2C,D; Movie 6). Expression of this construct only in muscle cells also shows that the effect of integrin on filopodia suppression is cell autonomous rather than due to contributions from tendon cells.

The finding that filopodia number was affected by both integrin loss and gain of function suggests an active role for integrin signaling in filopodia formation. To get further insights into this mechanism, we also analyzed the maximum length reached by each filopodia in our dataset. Maximum lengths of LT muscle filopodia follow an approximately exponential distribution (a high number of small filopodia and gradually fewer longer ones) (Fig. S3). This distribution is indicative of stochastic incorporation of actin to generate filopodial length rather than generation of a preferred length. We display this as a normalized cumulative frequency chart (Fig. 2E) to make the differences between conditions easily visible. Similar to the reduction in numbers of filopodia, the lengths of filopodia were also reduced as the embryo develops from stage 15 to stage 16 (Fig. 2E; the line shifts to the left, showing proportionally fewer long filopodia). The filopodia in *mys^XG43^* mutants at stage 15 were similar to controls, but at stage 16 the reduction in length of filopodia failed to occur (the stage 16 *mys^XG43^* line overlays that for the stage 15 controls). Conversely, when diβ was expressed in muscles, the maximum lengths distribution of stage 15 overlapped with the stage 16 control (Fig. 2E). This shows that dominant-active integrin signaling caused a premature reduction of the filopodia maximum length. This measure of filopodia length provides an independent quantification from filopodia number, hinting that integrin signaling may be affecting multiple points of actin regulation, affecting both the number and morphology of the resulting filopodia. We used live imaging of integrin and the membrane in an effort to discover the location of the integrin signaling. We detected faint levels of integrin within filopodia, intermediate levels at the base of some, but not all, filopodia, and high levels in dots separated from the filopodia (Fig. 2F–J). In 3D, the muscle tip is reminiscent of a fingertip pressed on a surface (the basal epidermis), with integrin adhesions at the fingerprint level (Fig. 2G–I) and filopodia at fingernail level (Fig. 2I–J). We did not detect any correlation between the integrin distribution and the presence or absence of filopodia, so it is not clear where signaling occurs.

### Git and Pak control filopodia formation downstream of integrins

We next investigated the signals and pathways downstream of integrins in LT muscles during attachment to the tendon. The protein Git has been implicated in the integration and transduction of various signals at the membrane, including integrins and receptor tyrosine kinases (Hoefen and Berk, 2006). Git recruits Pak to sites of focal adhesions, as part of its complex with Pak-interacting exchange factor (PIX), and at muscle ends (Bahri et al., 2009; Hoefen and Berk, 2006). Moreover, *Git* mutants have previously been reported to have defects in muscle patterning and targeting in ventral muscles (Bahri et al., 2009). In these mutants, the muscles target and attach inappropriately, suggesting a defect in myotube tips sensing tendon cells.

**Fig. 3.**
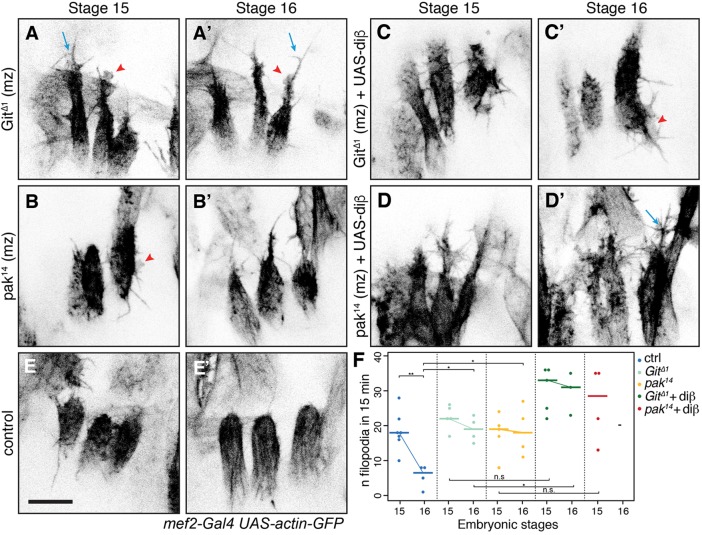
**Integrins act upstream of Git and Pak to suppress filopodia activity.** *Z* projections for *Git*^Δ*1*^ (A,A′), *pak^14^* (B,B′), diβ expression in *Git*^Δ*1*^ mz (C,C′) and *pak^14^* mz (D,D′) and control (E,E′). Blue arrows, branched filopodia; arrowheads, lamellipodia-like structures. Scale bar: 10 µm. (F) Quantification of filopodia number at dorsal LT muscle tips (Pak^14^+diβ stage 16, not enough values). Horizontal bars are the median. *P<0.025, **P<0.01 (see Table S1).

In embryos lacking both maternal and zygotic contributions of Git (*Git* mz), LT myotubes displayed a more elongated and pointed tip instead of the rounded shape observed in wild-type embryos both at stage 15 and 16 (Fig. 3A,E′), and the reduction in filopodia number at stage 16 failed to occur (Fig. 3F). As with loss of integrin, the filopodia appeared longer than in controls, but also more branched (Fig. 3A,A′, blue arrows). In addition, large protrusions, similar to lamellipodia, formed at muscle ends (Fig. 3A,A′, red arrowheads; Movie 7), which unfortunately prohibited an accurate quantification of filopodia length. A very similar phenotype was observed in embryos lacking both maternal and zygotic contributions of Pak (*Pak* mz) (Fig. 3B,B′; Movie 8). Thus, Git and its downstream effector Pak are also required to suppress filopodia at stage 16 and have an additional role in regulating the morphology of actin protrusions. The appearance of these larger protrusions when muscles lack Git or Pak suggests that there is normally a basal level of signaling through Git to suppress these structures. This is consistent with defects caused by the absence of Git in ventral muscles prior attachment, at stage 14–15 (Bahri et al., 2009).

To test whether Git and Pak act downstream of integrins, or via parallel pathways, we expressed diβ in embryos lacking Git or Pak. The ability of diβ to prematurely suppress filopodia at stage 15 was lost in the absence of Git or Pak (Fig. 3C,D,F; Movies 9,10). The number of filopodia in embryos deficient in Git or Pak and expressing diβ were somewhat elevated further than either *Git* or *Pak* alone (however, only one of the comparisons reached statistical significance). Diβ also exerts dominant negative effects on adhesion (Tanentzapf et al., 2006), which may allow increased filopodia in the absence of an inhibitory signal. Overall, we conclude that Git and Pak act downstream of integrin signaling to suppress filopodia.

### Conclusions

During morphogenesis, cells must simultaneously integrate several signals and adapt their morphology in coordination with their environment. During their attachment to tendon cells, myotubes need to recognize their target partner, stop elongating, start forming an adhesion and prepare the actomyosin structures that will produce muscle contractions in a short time. We showed that filopodia, which can be used to explore and identify tendon cells and are involved in myotube elongation, are actively suppressed during muscle attachment. Integrins, which accumulate at the myotube tip to form a strong adhesion with tendon cells, also provide a signal in parallel, acting through Git and Pak proteins. This is needed for the regulation of filopodia formation in order to ensure correct myotube arrest and adhesion to tendon cells. It is likely that multiple proteins within the cytoskeletal machinery are targeted by Git and Pak, possibly through direct phosphorylation or indirect regulation. Ena, the Scar/WAVE complex and fascin are all known actin regulators that are sensitive to phosphorylation state and could be such targets (Comer et al., 1998; Loureiro et al., 2002; Mendoza, 2013; Zanet et al., 2012; Zeng et al., 2017). To conclude, our results demonstrate that filopodia suppression occurs via integrin signaling within the myotube, providing a valuable new paradigm for integrin signaling in *Drosophila* and the roles of integrins in filopodia regulation.

## MATERIALS AND METHODS

### *Drosophila* stocks

Flies were raised and crossed at room temperature. The wild-type strain used was *white[1118]*. *Mef2-Gal4* was used to drive expression of these UAS constructs: w*; P{UAS-pGFP.Act88F}1-2 (BL9254), w*; *P{UAS-pGFP.Act5C}2-1* (BL 9258), w*; P{UAS-CD4-tdTomato} (BL35837) all from the Bloomington stock center, and UAS-diβ (Martin-Bermudo and Brown, 1999). The genomic rescue construct used in this study was: *βPS-GFP* (Klapholz et al., 2015). The mutant lines include: *mys[XG43]* (Bunch et al., 1992), *if[B4]* (Brown, 1994), *Git[Δ1]* (in this study) and *pak[14]* (Newsome et al., 2000; Bloomington stock center stock BL9123). *Git[Δ1]* and *pak[14]* germline clones were generated using *FRTG13 Git[Δ1]* and *FRT82B pak[14]* flies individually, essentially as described previously (Chou and Perrimon, 1996).

### Mutagenesis

The fly line carrying the P-element insertion *P{EPgy2}EY09254* was used to isolate mutations in *Git*. Mobilization of the P-element was achieved by using the immobile element *P[ry+ Δ2-3](99B)* as a transposase source (Robertson et al., 1988). The genotype of the transposase stock is *Sp/CyO; P[ry+ Δ2-3](99B)/TM6*. Single jump starter males of the genotype *P{EPgy2}EY09254/CyO; P[ry+ Δ2-3](99B)/+* were crossed to second chromosome balancer females. From each cross, three white-eyed male progeny were crossed individually to a deficiency *Df(2R)Stan2, FRT42D/CyO* to screen for lethality. The *Git* alleles were analyzed by PCR for mapping of lesions in the *Git* locus. The excision allele *Git[Δ1]* deletes 1472 bp including the ATG and the first exon (−520 to 952 relative to the first ATG of the *Git* gene).

### Live imaging of *Drosophila* embryos

Dechorionated embryos (washed in 50% bleach) were mounted on a glass-bottomed dish with heptane glue and covered with water. In some cases, the genotype of embryos was determined by the absence of YFP-marked chromosome balancer prior to and after the recording. Live imaging was performed on an inverted Leica TCS-SP5 equipped with a 63×1.4 NA Plan Apo oil immersion objective or an Olympus FV-1000 confocal laser scanning microscope equipped with a 60×1.3 NA Plan Apo oil immersion lens at room temperature. To follow filopodia movement, we visualized stage 15–17 embryos in the indicated genetic backgrounds. Time-lapse movies of LT tips in embryos were recorded by taking *z*-stacks of 5–7 *z* sections (0.7 µm spacing) at 15-s intervals for 15 min. For experiments, movies were recorded from three to five embryos per genotype, from different segments when recorded on the same embryos.

### Data analysis

The segmentation pipeline takes every *z*-stack acquired throughout an imaging session separately. It outputs filopodia segments that are then edited by a user, followed by an automatic linking step that assigns filopodia identities across time points. The segmentation pipeline consists of the following seven steps.

#### Pixel segmentation

We use the *slic* segmentation algorithm (Achanta et al., 2012) without enforced connectivity to turn the greyscale frame into a black-and-white segmented frame without blurring. We allow three labels to give the algorithm enough finesse to resolve filopodia and then consider every pixel that the algorithm assigns a label to as foreground.

#### Cell body identification

In order to identify cell bodies to mask the cell interior, we employ aggressive gaussian blurring (σ=5) together with adaptive thresholding with a large (999×999 pixels) gaussian-weighed neighborhood (van der Walt et al., 2014) together with an erosion operation with a 5-pixel disk as stencil. We then use this as a mask to remove parts of the skeleton that lie inside cell bodies.

#### Vesselness filter

We employ a modified version of the Frangi Vesselness measure that is popular for tracing blood vessels in MRI data (Frangi et al., 1998). This filter employs the eigenvalues of the Hessian, which describe the local curvature of the image signal. In particular, the smallest of the eigenvalues describes the local ‘saddleness’. We therefore calculate the eigenvalues λ_1_, λ_2_ and λ_3_ of the Hessian for each pixel in the output of the pixel segmentation and order them such that |λ_1_|<|λ_2_|<|λ_3_|. We then calculate the derived quantities *r*_*a*_ = |λ_2_|/|λ_3_|, 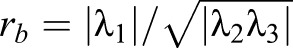 and 
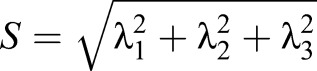
. They are then combined into the Vesselness measure

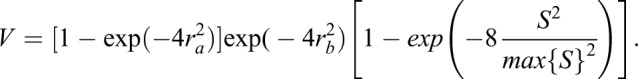


The exponential weights were chosen empirically such that filopodia in test movies were identified correctly.

#### Vesselness output segmentation

We again employ the slic segmentation algorithm without enforced connectivity, but a compactness parameter of 10 to identify bright connected lines in the Vesselness filter output and subsequently employ a binary closing step to heal broken lines.

#### Remove small objects

In this step, we remove small, connected regions containing less than 200 pixels (note that this step still operates on a *z*-stack of images, hence the rather high pixel number threshold), which stem from noise and not actual filopodia.

#### Skeletonization

We use the 3D skeletonization algorithm in Lee et al. (1994) to turn the resulting elongated patches into thin lines. We remove parts of the skeleton that are inside the cell bodies as detected in step 2.

#### Detection of connected line segments

We now label and iterate over all the connected regions of the skeleton. Each region has a collection of connected pixel coordinates (forming branched lines). We algorithmically split them into line segments and save them for subsequent manual editing and merging.

Following the automated extraction of filopodial line segments, we manually remove line segments that either were erroneously detected (due to e.g. noise in the image, or from parts of other structures in the field of view) or are part of filopodia stemming from cells other than the LT muscle cells. We also join line segments forming part of the same filopodium.

After the manual editing step, we automatically link and identify filopodia across time points. For each time point, we use the coordinates of all pixels contained in all filopodia over the preceding five time points. For each filopodium in the present time point, we then perform a next-neighbor search to find all previous filopodia that are close (with a cutoff radius of 25 pixels). For each of the candidate matches we calculate the path length difference measure *M*_*L*_=|*L*_*c*_−*L*_*p*_|/*max*{*L*_*c*_, *L*_*p*_} (where *L_c_* and *L_p_* are the path lengths of the current filopodium and the candidate match filopodium, respectively) and the average direction measure 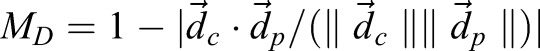 (where 
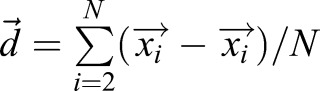
 is the average non-normalized direction vector of a filopodium). We then summarize these measure into a score *S*=0.46*M*_*L*_+0.6*M*_*D*_ (where the weights were manually adjusted to yield optimal results) and store the current filopodium and the match candidate together with the score. The pairs are then sorted by their score (lowest to highest) and iteratively linked until no more possible matches can be performed. Any leftover filopodia that were not matched to previously existing filopodia are considered to be newly created. After this step, we save the list of filopodia and their segment coordinates in each frame in which they exist and extract quantities such as the maximum filopodia length (over the lifetime of a filopodium) and the number of filopodia in existence in each movie.

## Supplementary Material

Supplementary information
