# Supplementary material for: Integrin signaling downregulates filopodia during muscle–tendon attachment
Source: J Cell Sci. 2018 Aug 16;131(16):jcs217133. doi: 10.1242/jcs.217133 (PMC6127725; doi:10.1242/jcs.217133)
Supplement: Supplementary information [file joces-131-217133-s1.pdf]

Table 1: Statistics

**Figure 2D:**		
n. filopodia	Non parametric Kruskal Wallis test:	0.0011
	post hoc conover test (significant if p < α/2):	
	ctrl st15 ~ ctrl st16	0.0016
	ctrl st16 ~ mys^{XG43} st16	0.0145
	ctrl st15 ~ *UAS-dib* st15	0.0027
**Figure 2E:**		
Maximum length	Non parametric Kruskal Wallis test:	0.037
	post hoc conover test (significant if p < α/2):	
	ctrl st15 ~ ctrl st16	0.0018
	ctrl st16 ~ mys^{XG43} st16	0.0157
	ctrl st15 ~ *UAS-dib* st15	0.0227
**Figure 2 F:**		
GFP intensity	Ttest (two tails)	
	Background ~ filopodia	$9.77x10^{-07}$
	Filopodia ~ filopodia base	0.00011
	Filopodia base ~ integrin spots	$1.87x10^{-09}$
**Figure 3:**		
n. filopodia	Non parametric Kruskal Wallis test:	0.00561
	post hoc conover test (significant if p < α/2):	
	ctrl st15 ~ ctrl st16	0.0080
	ctrl st16 ~ $Git^{\Delta 1}$ st16	0.0132
	ctrl st16 ~ pak^{14} st16	0.0115
	$Git^{\Delta 1}$ st15 ~ $Git^{\Delta 1}$+*UAS-dib* st15	0.0446
	$Git^{\Delta 1}$ st16 ~ $Git^{\Delta 1}$+*UAS-dib* st16	0.0137
	pak^{14} st15 ~ pak^{14}+*UAS-dib* st15	0.0451
	pak^{14} st16 ~ pak^{14}+*UAS-dib* st16	NA

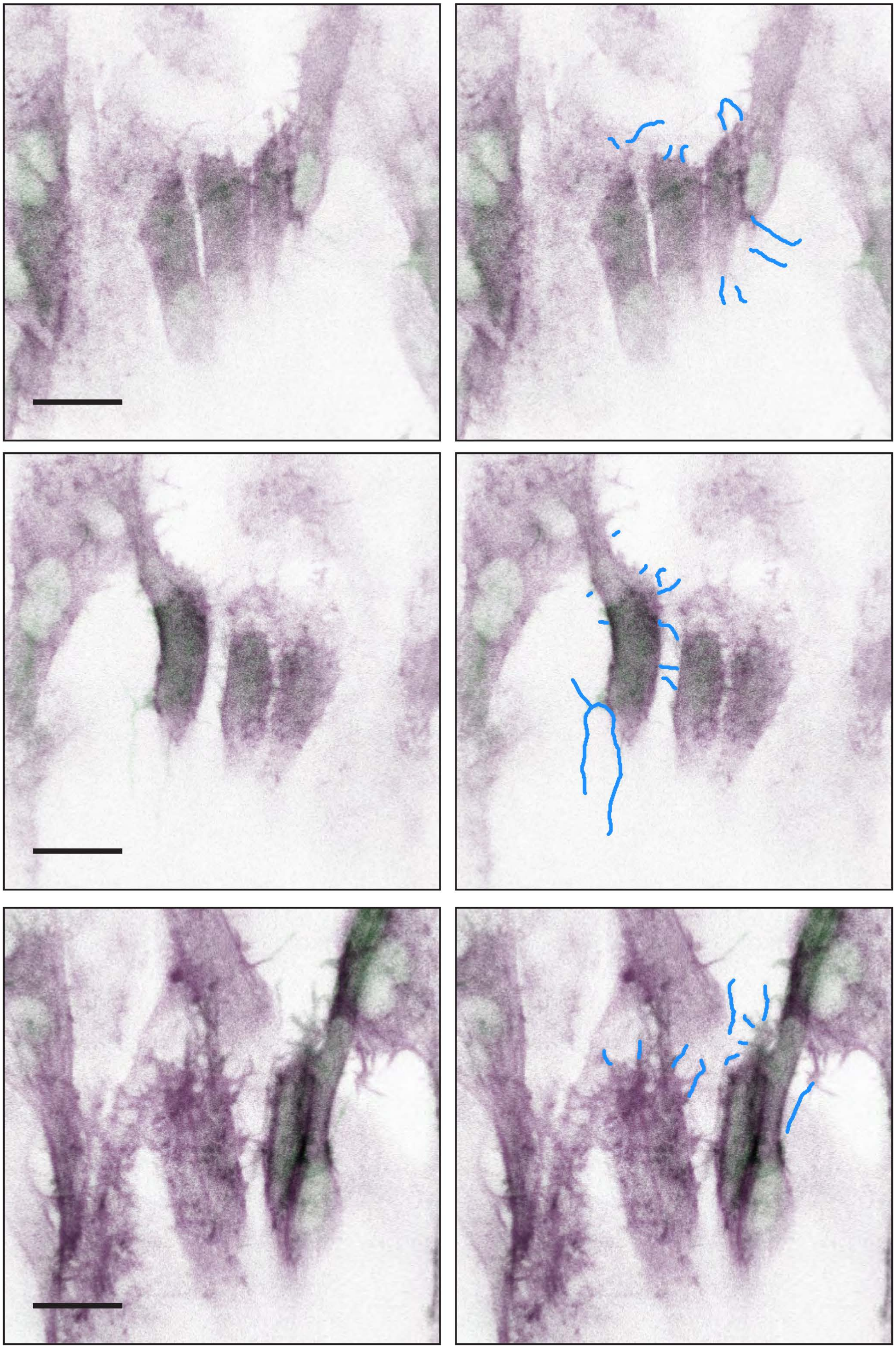

Supplementary figure 1: Semi-automated image segmentation results

Confocal still images from timelapse movies taken of (A) and (B) control embryos at stage 16, and (C) a pak-mz UAS-diβ embryo at stage 16. The left panel displays the confocal image stack, coloured by z-slice, magenta to dark green. The right panels display the final segmented filopodia (blue overlays) projecting from myotubes. Scale bar: 10 μm.

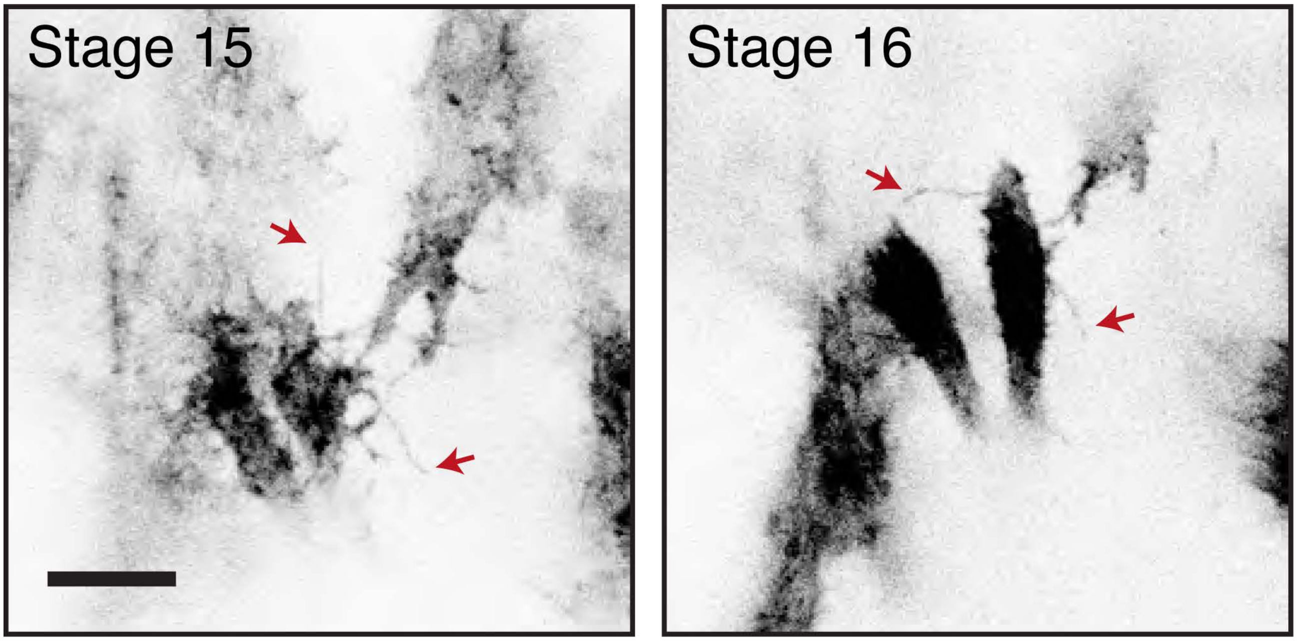

Supplementary figure 2: Filopodia at LT tips in inflated mutants

Still images from timelapse movies showing zygotic αPS2 mutant *inflated*^{B4}. Although actual reconstruction of filopodia was not possible in this genotype, the movies show that there is no reduction in the number of filopodia (red arrows) at stage 16 compared to control embryos (see control in figure 2 and Movie 5). Scale bar: 10 μm.

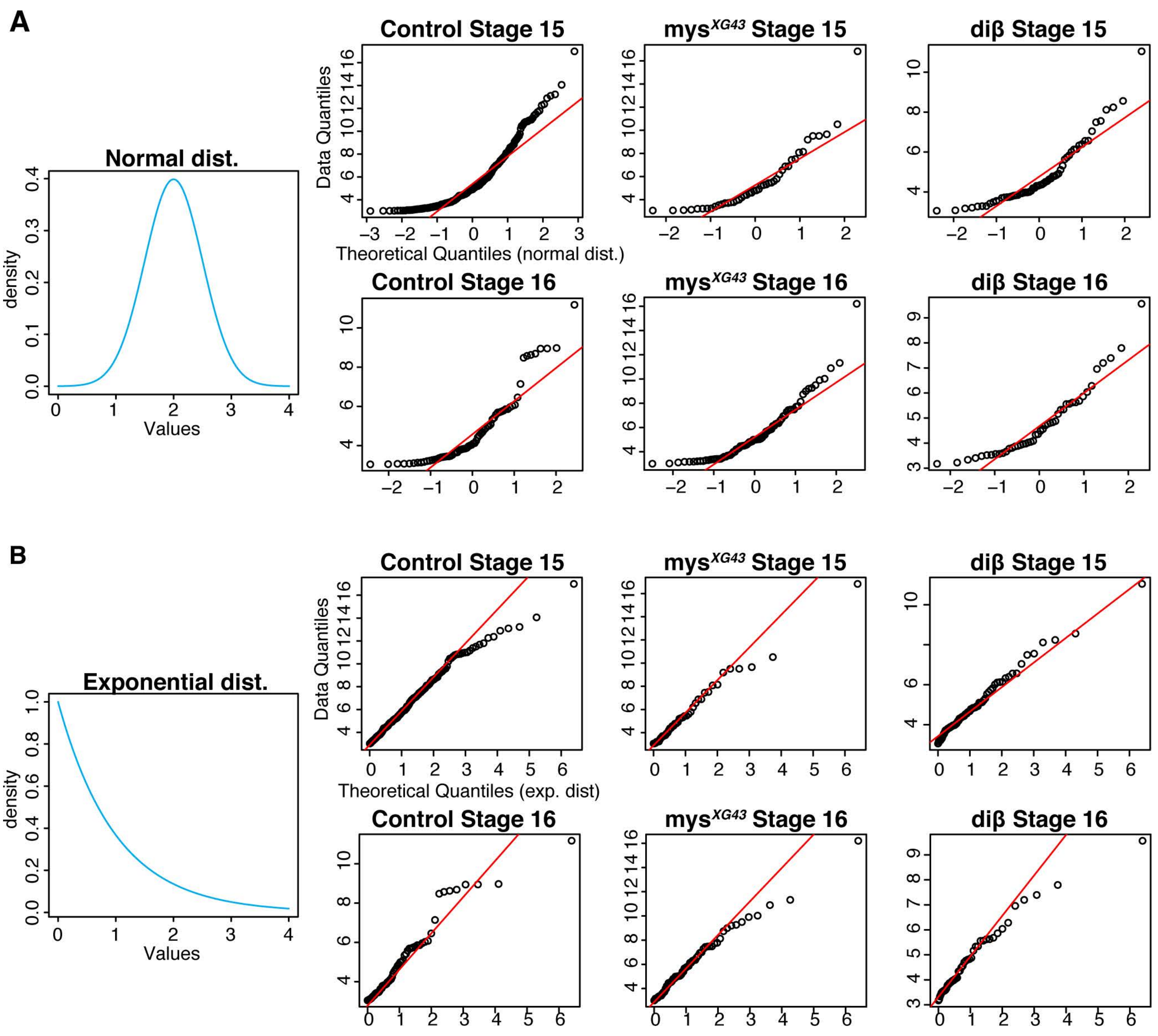

Supplementary figure 3: Filopodia maximum lengths follow an exponential distribution

Quantile-Quantile (QQ) plots showing the distribution of the values of maximum length reached by each filopodia compared to a normal distribution (A, blue curve) and an exponential distribution (B, blue curve). Filopodia maximum length values (Y axis), are organized into quantiles (groups of equal numbers of values (c.f. percentile), indicated in the graph by each black circle) and plotted against values on the X axis issued from a theoretical normal distribution (A) and a theoretical exponential distribution (B). When the experimental and theoretical values follow the same distribution, the quantiles are aligned along the fitting (red) line. Maximum length values do not fit a normal distribution as quantiles are spreading away from the fitting line (A). (B) For all conditions, data follow an exponential distribution except for the longest filopodia, which slightly deviate from the exponential distribution. This could be due to a bias in the imaging/reconstruction process or originate from a limiting factor in the elongation of filopodia.

Journal of Cell Science • Supplementary information

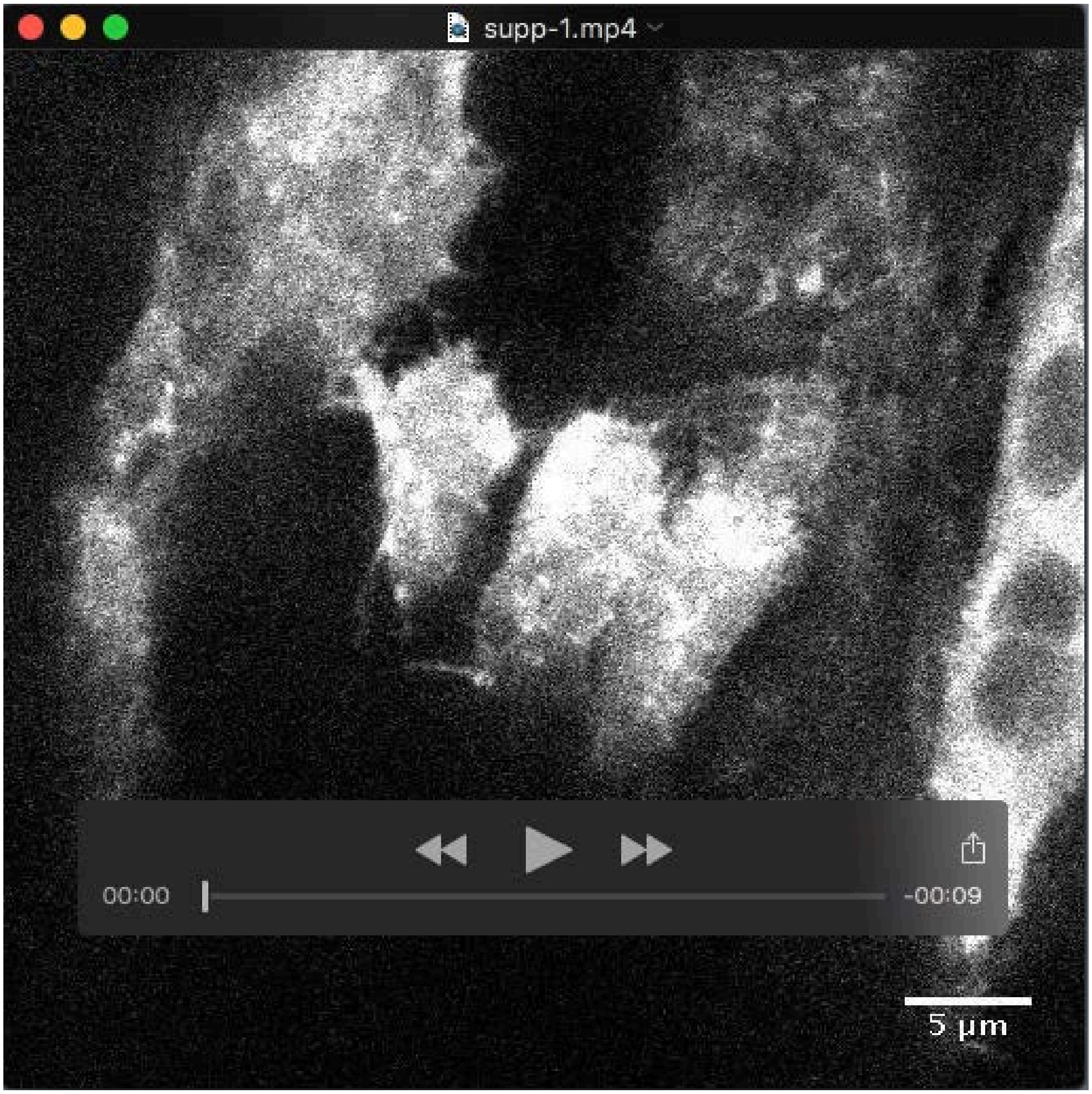

Movie S1: control movie of *mef2-Gal4 UAS-GFPactin* timelapse imaging of dorsal LT muscle tip at stage 15.

Journal of Cell Science • Supplementary information

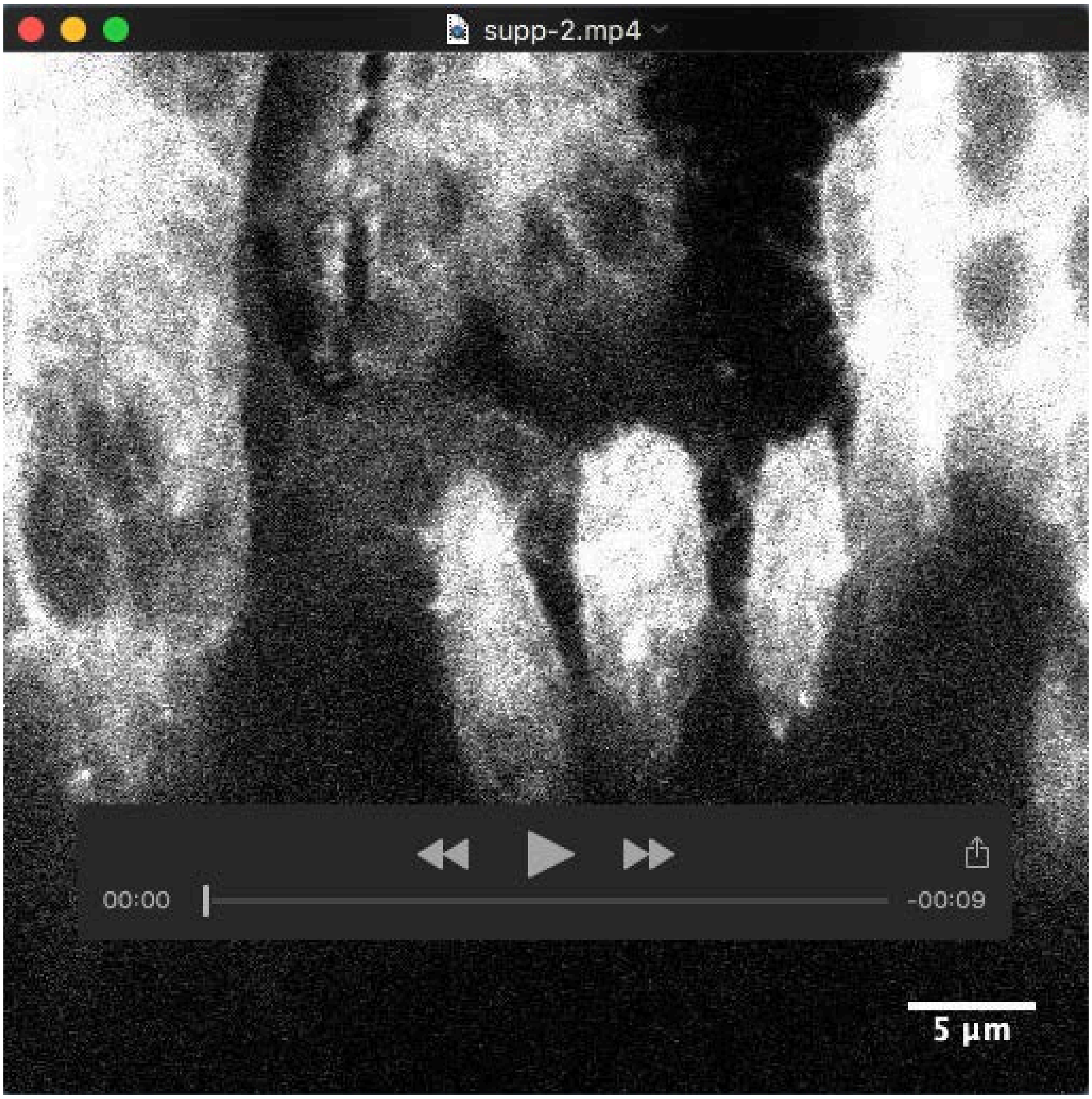

Movie S2: control movie stage 16.

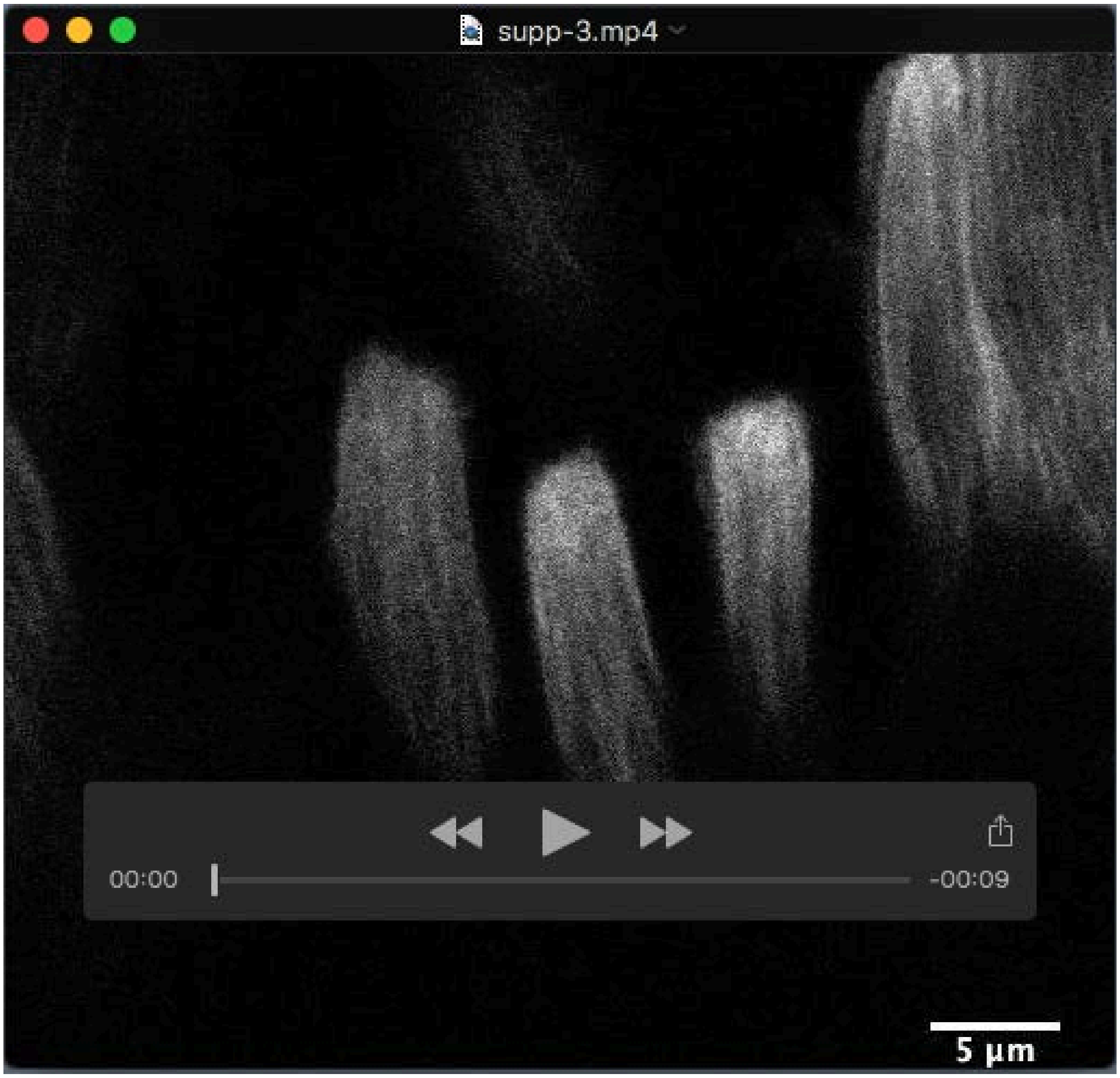

Movie S3: control movie stage 17.

Journal of Cell Science • Supplementary information

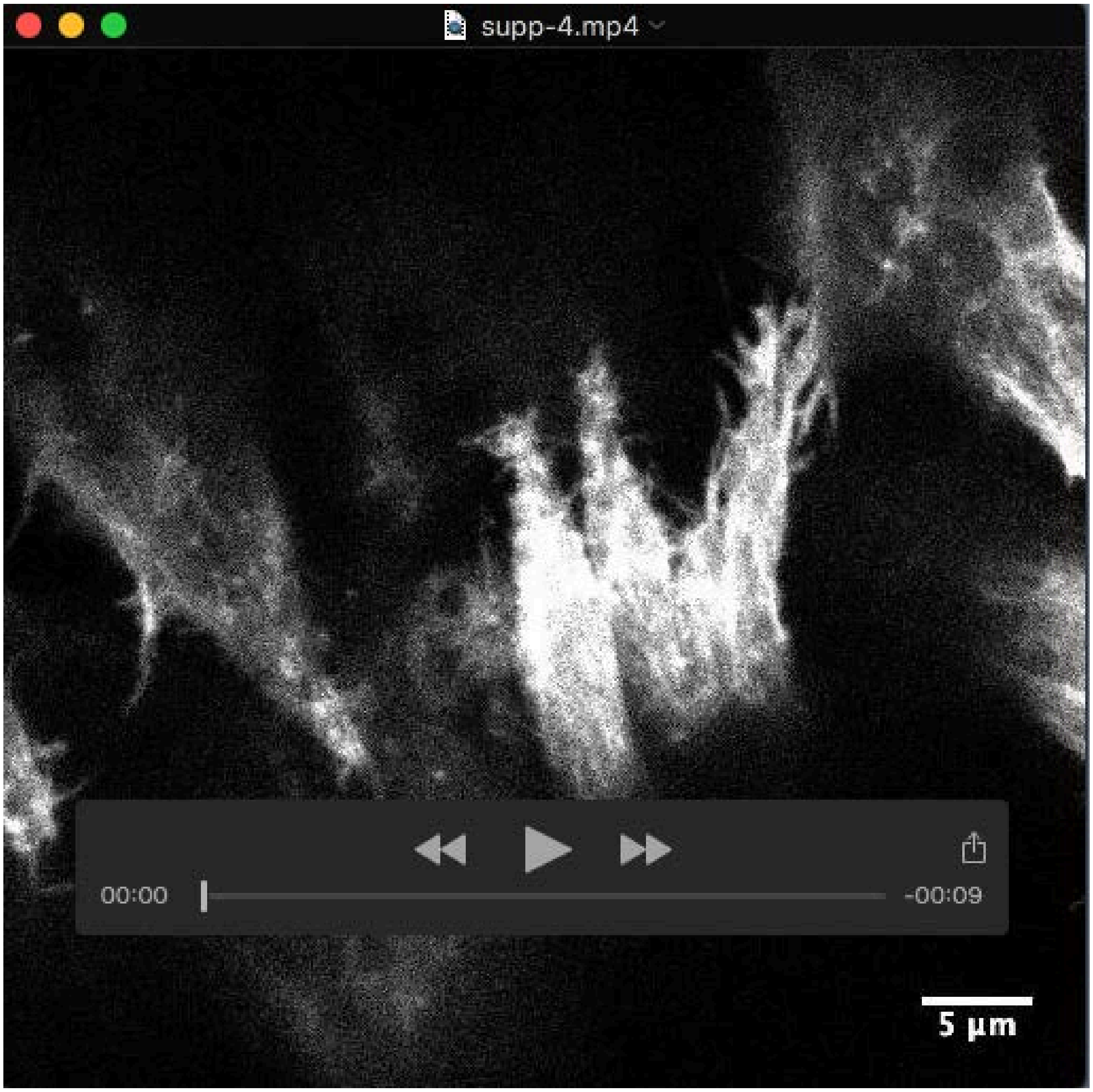

Movie S4: *mys*^{XG43} mutant at stage 16.

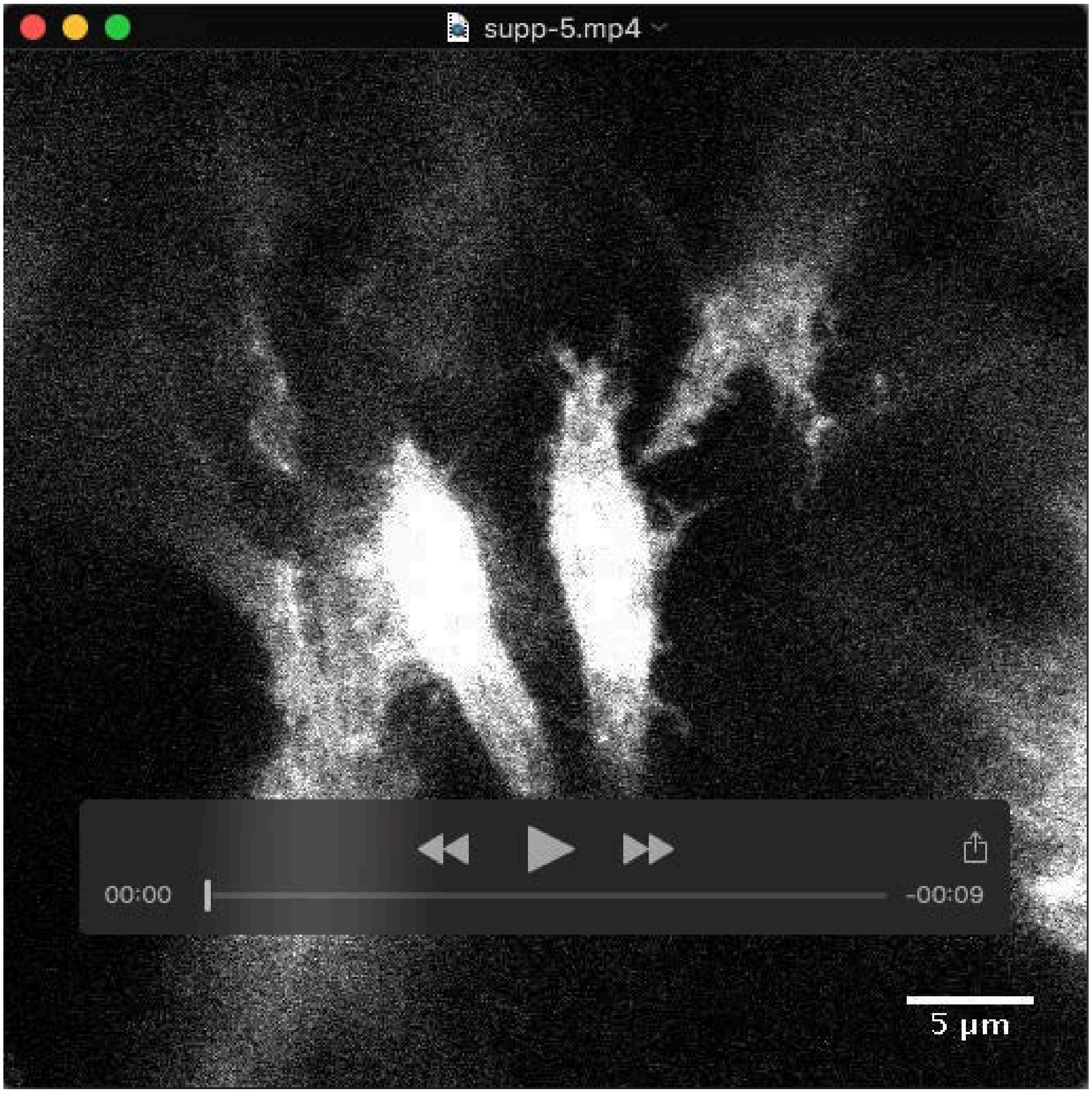

Movie S5: *if*^{B4} mutant at stage 16.

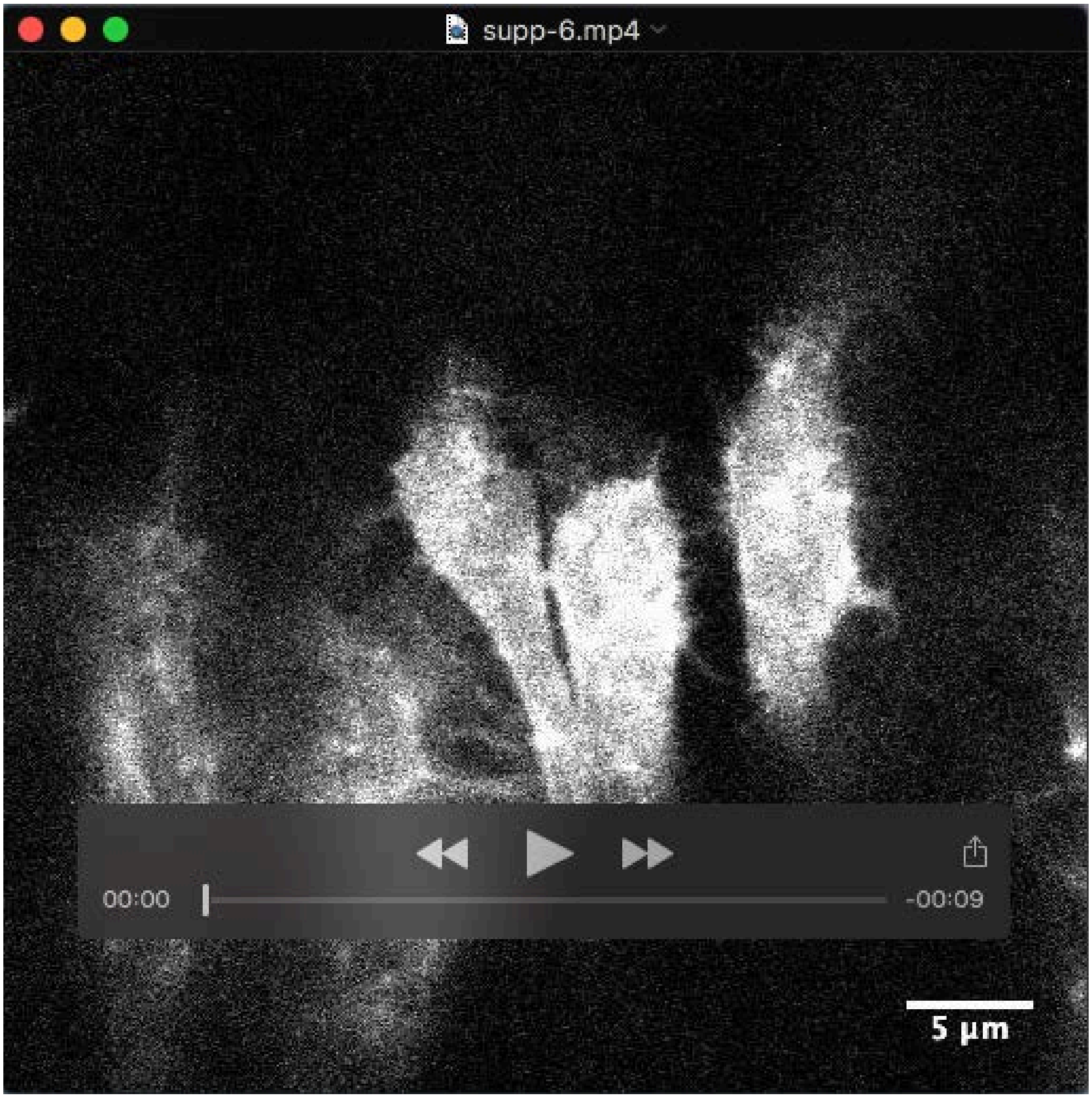

Movie S6: Overexpression of UAS-diβ in muscles at stage 15.

Journal of Cell Science • Supplementary information

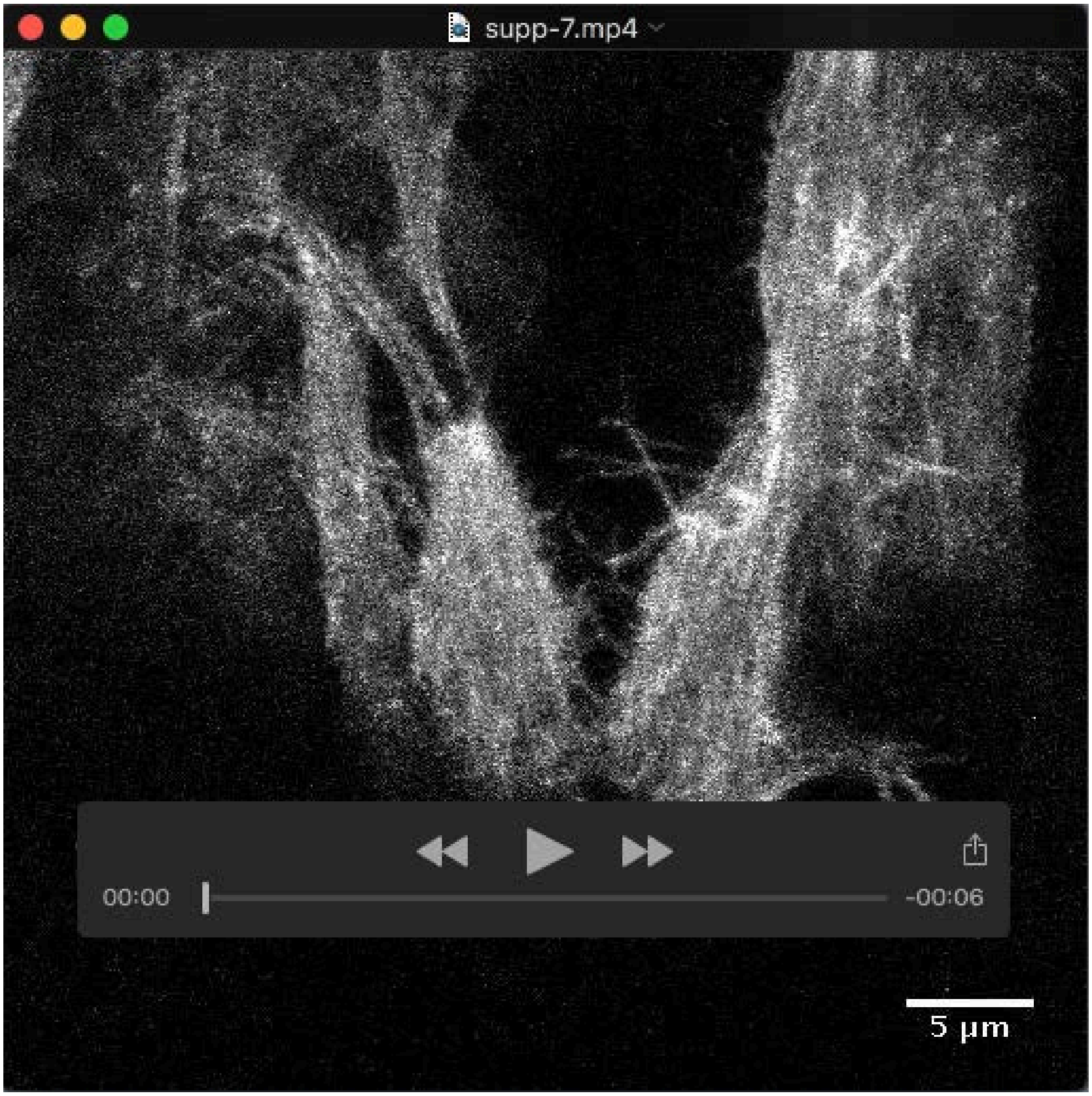

Movie S7: *Git*$^{\Delta 1}$ mz mutant stage 16.

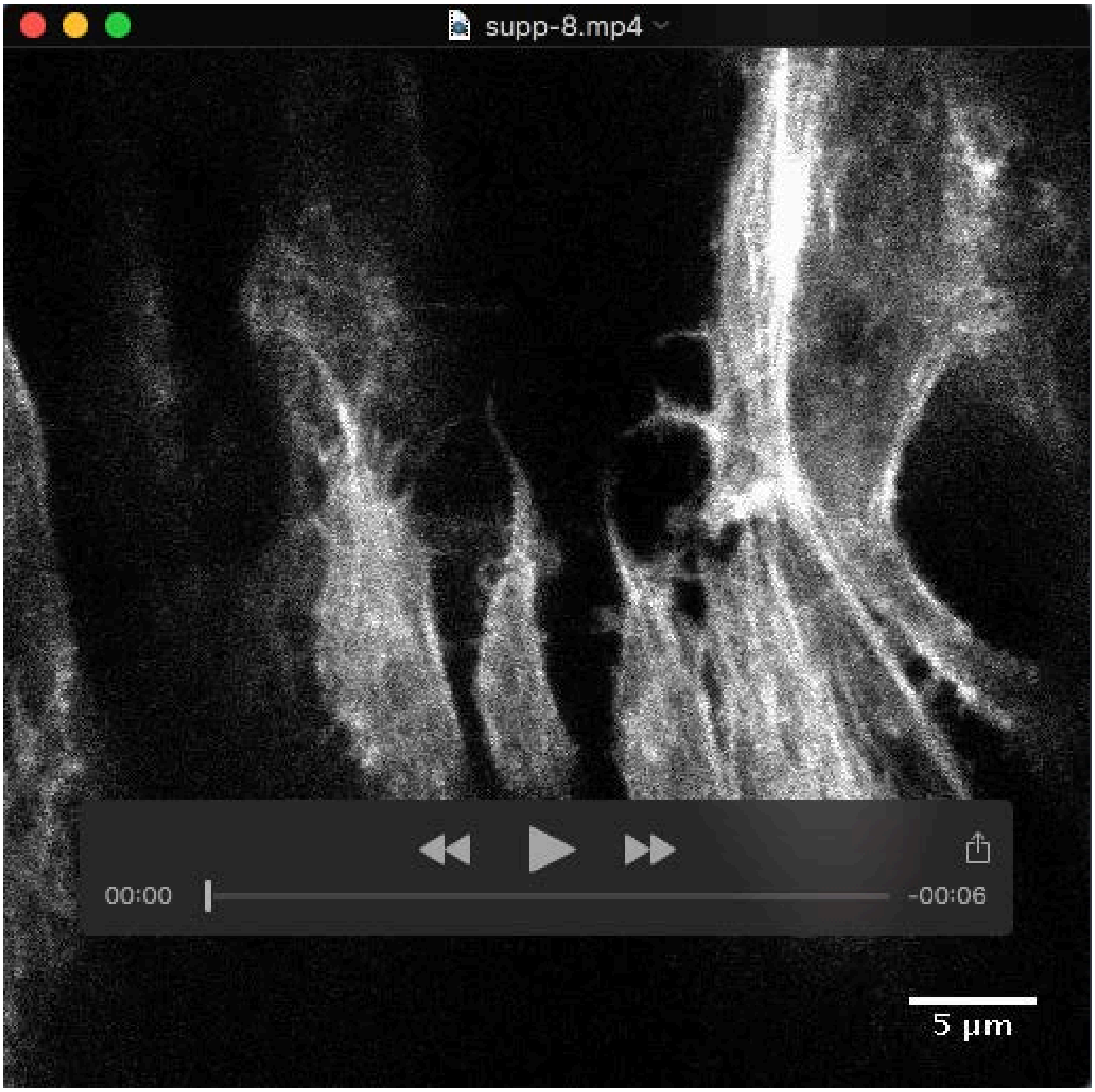

Movie S8: *pak*^{14} mz mutant stage 16.

Journal of Cell Science • Supplementary information

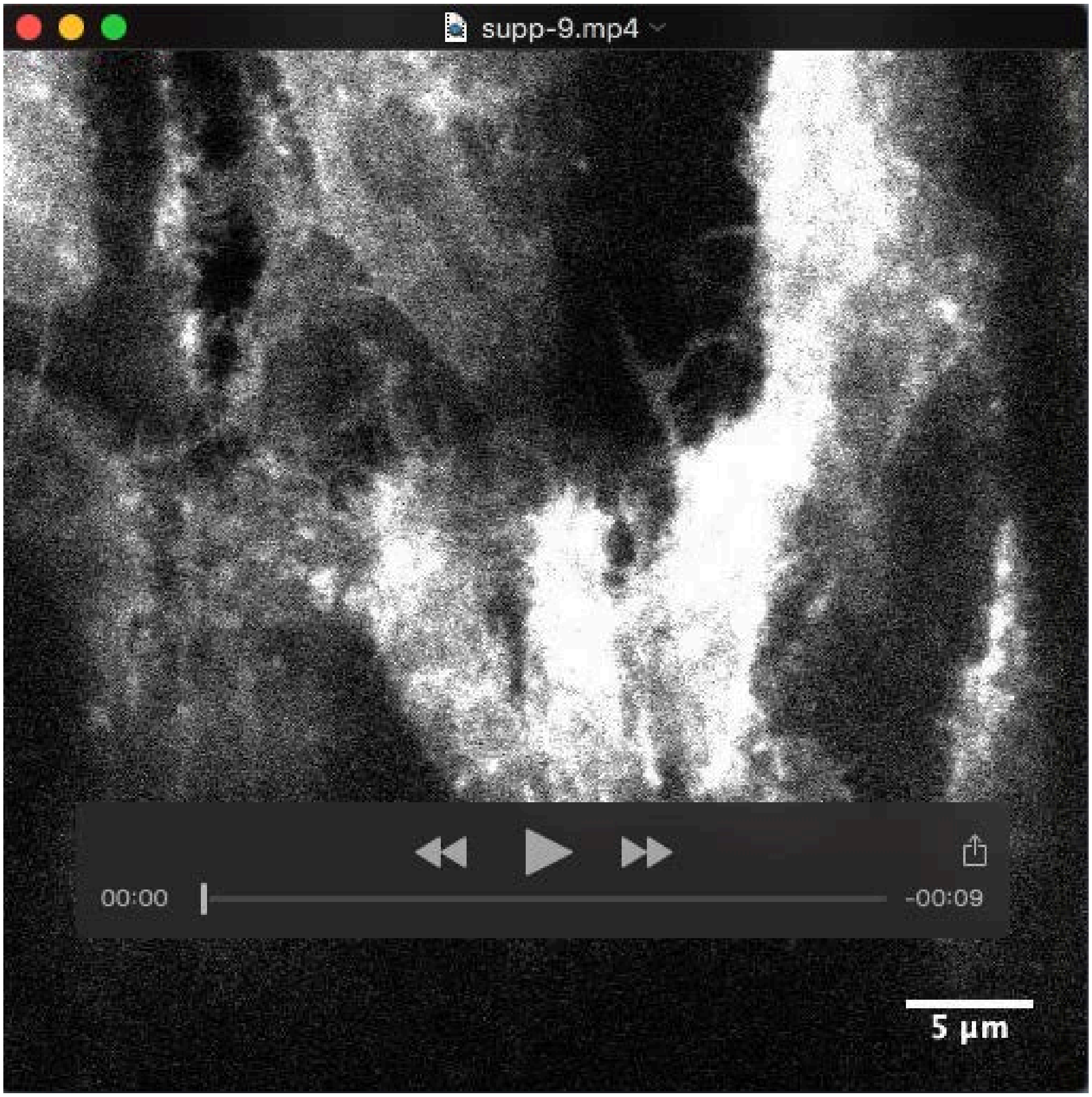

Movie S9: *Git*$^{\Delta 1}$ mz with UAS-diβ expression stage 15.

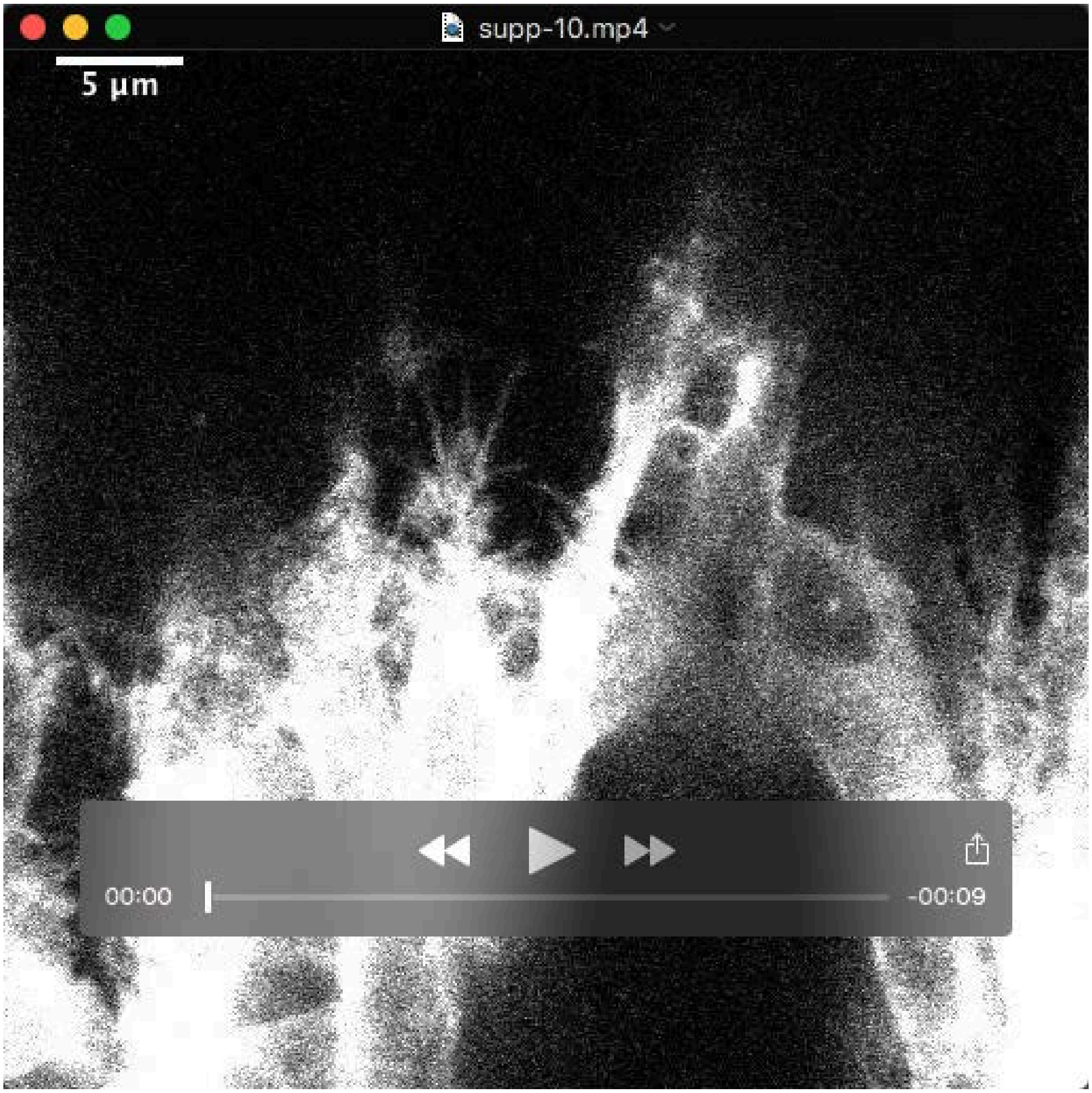

Movie S10: *pak*^{14} mz with UAS-diβ expression stage 15.
